# Bacterial Community Mapping of the Mouse Gastrointestinal Tract

**DOI:** 10.1371/journal.pone.0074957

**Published:** 2013-10-07

**Authors:** Shenghua Gu, Dandan Chen, Jin-Na Zhang, Xiaoman Lv, Kun Wang, Li-Ping Duan, Yong Nie, Xiao-Lei Wu

**Affiliations:** 1 College of Engineering, Peking University, Beijing, China; 2 Kunming Medical University, Chengong, Kunming, Yunnan, China; 3 Yunnan University of Traditional Chinese Medicine, Chengong, Kunming, Yunnan, China; 4 Peking University Third Hospital, Peking University, Beijing, China; University of Utah, United States of America

## Abstract

Keeping mammalian gastrointestinal (GI) tract communities in balance is crucial for host health maintenance. However, our understanding of microbial communities in the GI tract is still very limited. In this study, samples taken from the GI tracts of C57BL/6 mice were subjected to 16S rRNA gene sequence-based analysis to examine the characteristic bacterial communities along the mouse GI tract, including those present in the stomach, duodenum, jejunum, ileum, cecum, colon and feces. Further analyses of the 283,234 valid sequences obtained from pyrosequencing revealed that the gastric, duodenal, large intestinal and fecal samples had higher phylogenetic diversity than the jejunum and ileum samples did. The microbial communities found in the small intestine and stomach were different from those seen in the large intestine and fecal samples. A greater proportion of Lactobacillaceae were found in the stomach and small intestine, while a larger proportion of anaerobes such as Bacteroidaceae, Prevotellaceae, Rikenellaceae, Lachnospiraceae, and Ruminococcaceae were found in the large intestine and feces. In addition, inter-mouse variations of microbiota were observed between the large intestinal and fecal samples, which were much smaller than those between the gastric and small intestinal samples. As far as we can ascertain, ours is the first study to systematically characterize bacterial communities from the GI tracts of C57BL/6 mice.

## Introduction

The adult mammalian gastrointestinal (GI) tract is home to microorganisms with the number around 10 times greater than the total number of mammalian somatic and germ cells [1]. Host–microbe interactions are now regarded as essential to many aspects of normal ‘mammalian’ physiology, ranging from metabolic activity to immune homeostasis [2]. Recent studies on GI microbiota confirmed that a balance in GI microbial communities is crucial for host health maintenance; perturbation of this microbial composition has been hypothesized to be involved in a range of diseases outside the gut, such as diabetes [3], obesity [4], fatty liver[5], inflammatory bowel diseases [6], anxiety [7] and even cancer [8]. Although increasing research has been performed on mammalian gastrointestinal tract microbial ecology, most of the samples used in these studies were from the feces. Consequently, our understanding of the characteristic microbiota in different sections along with the GI tract is still very limited, especially for C57BL/6 mice, which are one of the most commonly used animals for studying gut microbiota related diseases [9]. Because the comprehensive characterization of normal mouse GI tract microbial communities is a critical prerequisite to understanding and predicting alterations in these communities in relation to disease, we conducted a study to characterize the GI tract microbiota of specific pathogen free (SPF) C57BL/6 mice using a recently developed high-throughput pyrosequencing approach.

## Materials and Methods

### Animals and sample collection

Six male SPF C57BL/6 mice aged 10 weeks were used in this study. All animal care procedures were approved by the Institutional Animal Care and Use Committee of Peking University prior to initiation of the experiment. All mice were housed in one cage in a standard animal laboratory with a 12 h light–dark cycle and were fed with a standard diet. Commercial mouse chow (Academy of Military Medical Sciences, jun2007-005) and water were autoclaved before use. Feces were collected in advance of all experimental procedures. All mice were transferred to fresh sterilized cages and the feces were collected within two hours from the cages. Mice were then euthanized, before the contents of the stomach, duodenum, jejunum, ileum, cecum and colon were sampled, weighed and immediately frozen in liquid nitrogen. After the samples (42 in total) were thoroughly frozen, they were stored at −80°C until DNA extraction. The mean lengths of the murine small intestine (including duodenum, jejunum and ileum) and murine large intestine (including cecum and colon) were 42.5 and 11.3 cm, respectively. The murine jejunum is defined as the terminal transverse part of the murine small intestine.

### DNA Extraction, PCR amplification, amplicon quantization, pooling, and pyrosequencing

Total genomic DNA from each sample (100 mg) was extracted using the QIAamp DNA Stool Mini Kit according to the manufacturer's instructions. A region of about 180 bp, in the 16 S rRNA gene and covering the V3 region, was selected to construct a community library through tag pyrosequencing. The broadly conserved primers, 340F (5′-CCTACGGGAGGCAGCAG-3′) and 533R (5′-TTACCGCGGCTGCTGGCAC-3′), containing the A and B sequencing adaptors (454 Life Sciences) were used to amplify this region. In addition, these primers also contained an 11 nt barcode sequence that allowed for multiple samples to be analyzed in a single sequencing run. The PCRs were carried out in triplicate using 20 µl reactions with 0.6 mM each primer,10–50 ng of template DNA, 4 µl of the PCR reaction buffer and 2.5 U of Phusion DNA Polymerase. The amplification program consisted of an initial denaturation step at 94°C for 4 min, followed by 22 cycles, where 1 cycle consisted of 94°C for 10 s (denaturation), 55°C for 10 s (annealing) and 72°C for 15 s (extension), and a final extension of 72°C for 10 min. Negative controls were always performed to verify the lack of Taq performance without the DNA template. Replicate PCR products of the same sample were mixed within a PCR tube. They were then visualized on agarose gels (2% in TBE buffer) containing ethidium bromide, and purified with a DNA gel extraction kit (Axygen, China). Prior to sequencing, the DNA concentration of each PCR product was determined using a Quant-iTPicoGreen double stranded DNA assay (Invitrogen, California, USA) and was quality controlled on an Agilent 2100 bioanalyzer (Agilent, USA). Following quantization, the amplicons from each reaction mixture were pooled in equimolar ratios based on concentration and subjected to emulsion PCR to generate amplicon libraries, as recommended by 454 Life Sciences [10]. Amplicon pyrosequencing was performed from the A-end using a 454/Roche A sequencing primer kit on a Roche Genome Sequencer GS FLX Titanium platform at Majorbio Bio-Pharm Technology, Shanghai, China.

### Data analysis

Pyrosequencing reads with more than one ambiguous nucleotide or within correct barcodes or primers were removed and excluded from further analysis. Sets of sequences with ≥97% identity were defined as an Operational Taxonomic Unit (OTU). OTUs were assigned to a taxonomy using the Ribosomal Database Project (RDP) Naive Bayes classifier [11]. Representative sequences from each cluster were aligned with the PyNAST aligner [12] to the greengenes core set in QIIME [13]. A phylogeny was constructed within QIIME using FastTree [14]. Rarefaction curves, alpha diversity, and beta diversity calculations were also performed using QIIME. Phylogenetic diversity (PD) and Shannon diversity index (SI) were estimated to evaluate the ecological diversity of microbiota from each sample. SI is a quantitative measure that reflects how many different types (such as species) there are in a dataset, and simultaneously takes into account how evenly the basic entities (such as individuals) are distributed among those types. The value of a diversity index increases both when the number of types increases and when evenness increases. But the interpretation is hindered by uncertain species definitions and the lack of a statistical framework for comparing values. In contrast to SI, phylogenetic diversity (PD) takes into account the taxonomic breadth of samples without relying on morphotaxa, species or sequence-type designations [15]. To analyze the relationships between samples, dual hierarchal dendrograms were calculated, based on bacterial composition information at taxonomic levels. An analysis was performed with the NCSS 2007 software using weighted pair clustering which was based upon Manhattan distance measurements. The similarity among the microbial communities was determined using UniFrac analysis [16] in which weighted and unweighted principal coordinate analysis (PCoA) were performed. The OTU network was constructed by QIIME and visualized using Cytoscape [17] to map gut microbial community composition and structure onto the mouse GI tract, thereby complementing phylogeny-based microbial community comparisons. These analyses were used to bin 16S rRNA V3 gene sequences into OTUs and to display microbial genera partitioning across mouse GI tracts. OTUs and each sample were designated as nodes in a bipartite network, in which OTUs are connected to the samples in which their sequences were found. A spring-embedded algorithm was used to cluster the OTUs and samples.

### Statistical analysis

Changes in bacterial abundance were compared using repeated measures ANOVA analysis with the Tukey's honestly significant difference (HSD) post hoc test. Relationships between sequences and diversity and coverage were examined by Pearson's correlation. Statistical analyses were performed using Graohoad prism Program (version5.0.1, Graphpad software Inc., San Diego,CA,USA). Significance was accepted at P<0.05.

## Results

### Diversity of the bacterial community along the mice GI tract

After removed reads containing incorrect primer or barcode sequences and sequences with more than one ambiguous base, a total of 283,234 valid reads were obtained from the 42 samples through 454 pyrosequencing analysis. Each sample was covered by an average of 6743 reads (Table S1 in File S1). Good's coverage of all samples averaged 96.4±1.6% (mean±s.d., ranging from 91% to 96%) (Table S1 in File S1). The individual rarefaction curves tended to approach the saturation plateau except in one of the duodenal samples (Figure S1 in File S1). No significant correlation (Pearson's correlation, *P*>0.2) was found between the number of reads per sample, the number of OTUs, and the estimated number of OTUs.

**Figure 1:**
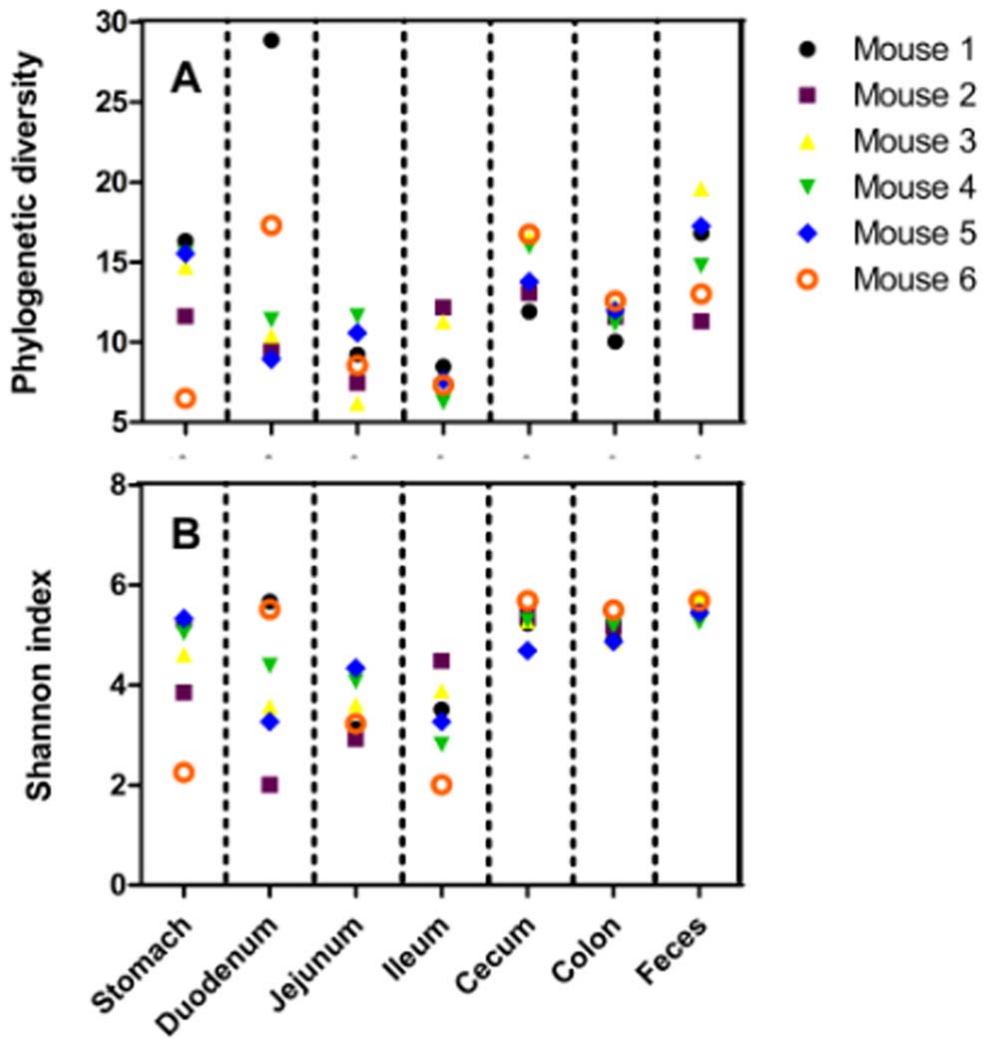
Alpha diversity. (A) phylogenetic diversity (PD) and (B) Shannon diversity of each GI site from the six mice.

PD and SI were estimated to evaluate the ecological diversity of microbiota from each sample. Generally, jejunal and ileal samples had the lowest diversity, while samples from cecum, colon, and feces had the highest PD (Figure 1A) and SI (Figure 1B) values. In addition, the PD and SI values of gastric and duodenal samples showed much higher inter-mouse variation than those from other samples.

### Changes in bacterial community structure along the mouse GI tract

**Figure 2:**
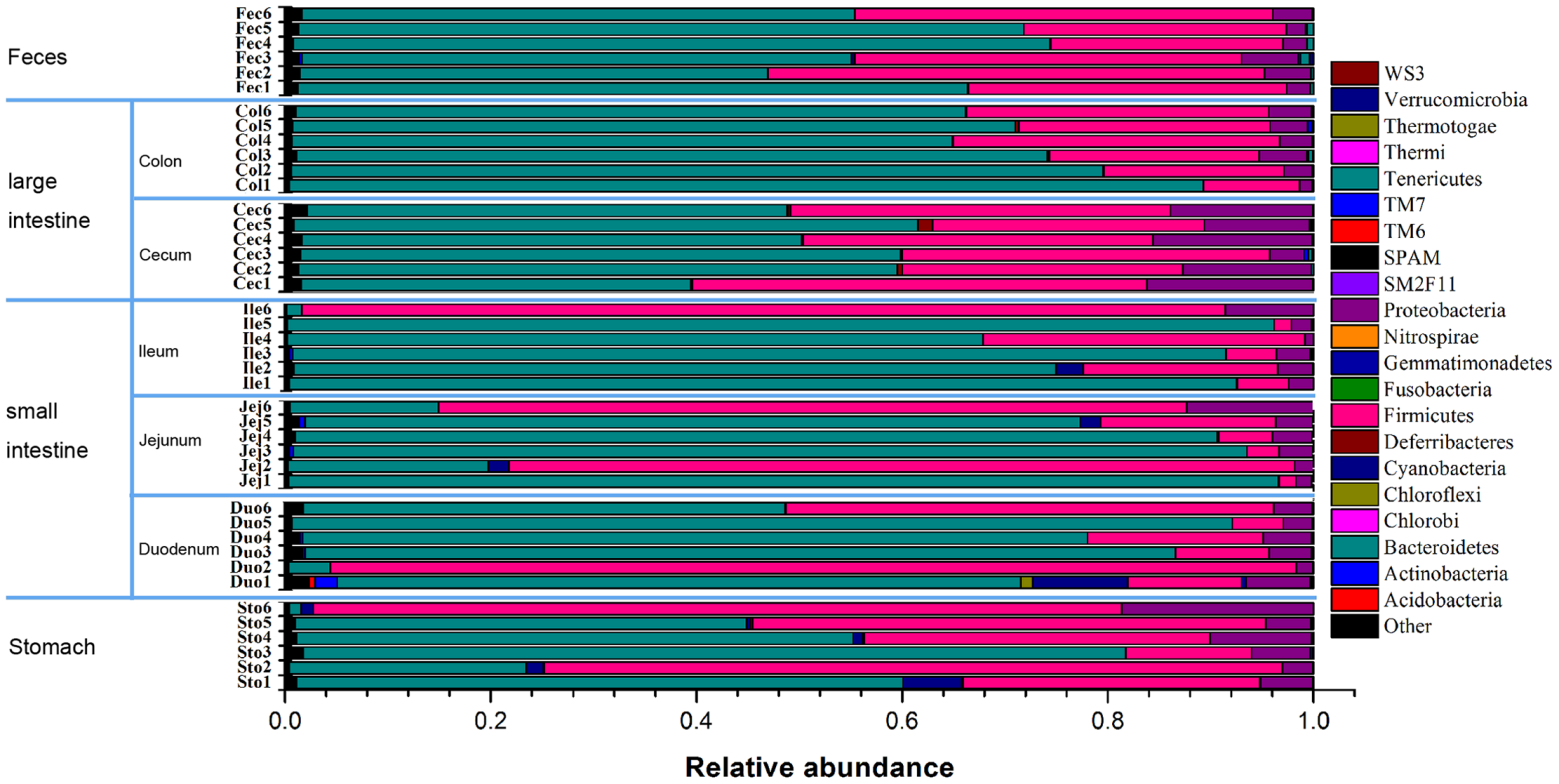
Relative abundance of sequences belonging to different bacterial phyla. Sequences that could not be classified into any known group were assigned as ‘Unknown’. Sto: Stomach samples; Duo: Duodenum samples; Jej: Jejunum samples; Ile: Ileum samples; Cec: Cecum samples; Col: Colon samples; Fec: Feces samples. The number following the abbreviations stands for the mouse number. For example, Cec1, Cec2, Cec3, Cec4, Cec5, and Cec6 stands for the Cecum sample from the 1^st^, 2^nd^, 3^rd^, 4^th^, 5^th^ and 6^th^ mouse.

Taxonomically, 21 different bacterial phyla or groups were identified (Figure 2). The majority of the sequences obtained belonged to Bacteroidetes (61.94%) and Firmicutes (30.55%) with the rest distributed among Proteobacteria (5.39%), Cyanobacteria (0.63%), Tenericutes (0.165%), Actinobacteria (0.13%), Deferribacteres (0.10%) and unclassified bacteria (0.95%). However, only Bacteroidetes, Firmicutes and Proteobacteria were found in all samples. Among the 7 GI sites, the duodenum harbored most of the phyla and groups, including the duodenum-unique Chlorobi, Chloroflexi, Nitrospirae, SM2F11, SPAM, TM6 and WS3 groups.

**Figure 3:**
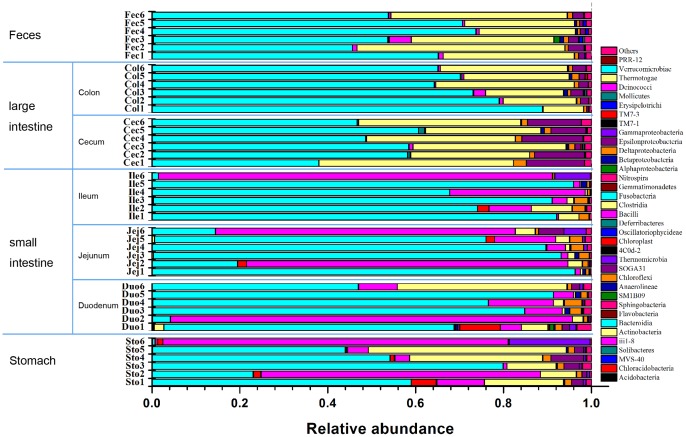
Relative abundance of sequences belonging to different bacterial Class. Sto: Stomach samples; Duo: Duodenum samples; Jej: Jejunum samples; Ile: Ileum samples; Cec: Cecum samples; Col: Colon samples; Fec: Feces samples. The number following the abbreviations stands for the mouse number. For example, Cec1, Cec2, Cec3, Cec4, Cec5, and Cec6 stands for the Cecum sample from the 1^st^, 2^nd^, 3^rd^, 4^th^, 5^th^ and 6^th^ mouse.

**Figure 4:**
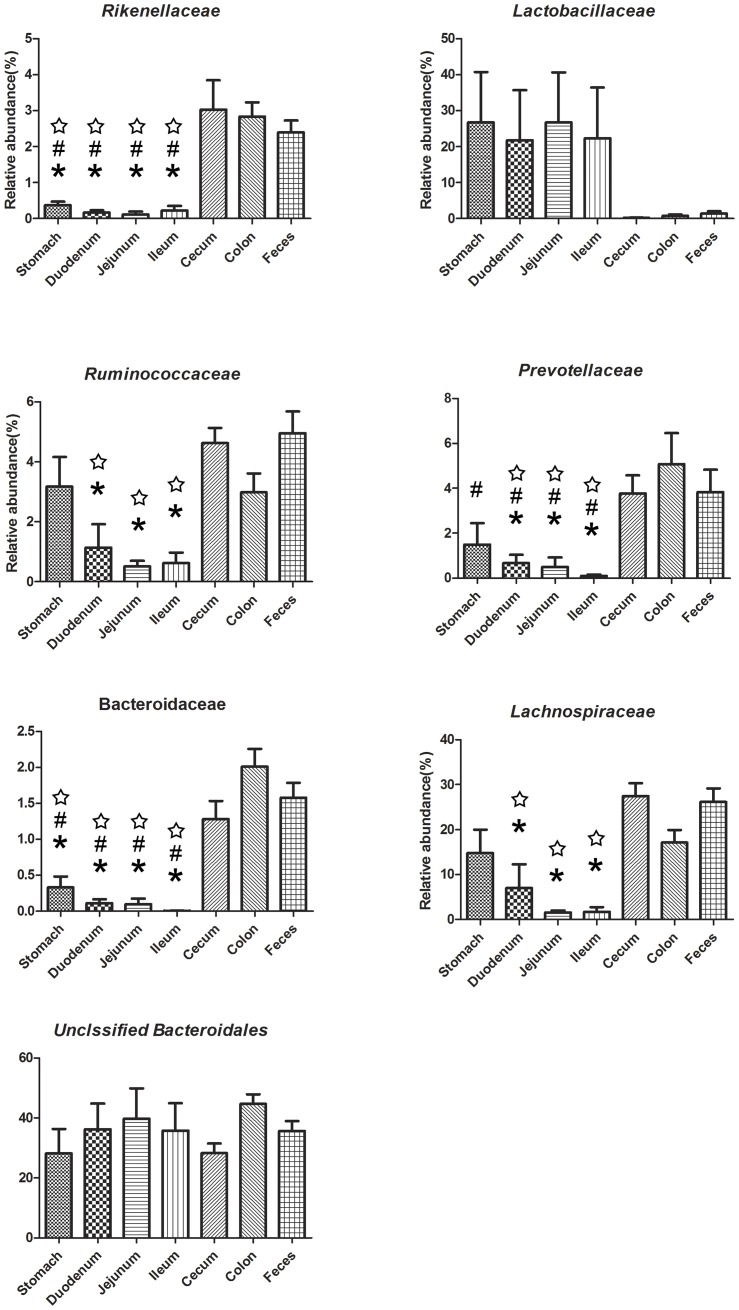
Relative abundance of sequences belonging to different bacterial Class. (☆, P<0.05, compared to Cecum; #, P<0.05, compared to Colon; *, P<0.05, compared to Feces, by Tukey's honestly significant difference (HSD) post hoc test).

While the bacterial community structure varied from different anatomical region along the mice GI tract. At the phylum level, the relative abundance of Proteobacteria was significantly higher (*P*<0.05) in cecum than that in other sites except for the stomach(Figure S2 in File S1). There is no significant difference along GI tract of Bacteroidetes, Firmicutes and other phyla or groups. At the class level, Bacteroidia (belonging to Bacteroidetes) dominate the GI tract, and there is no significant different along the GI tract (Figure S2 in File S1). The relative abundance of Bacilli (belonging to Firmicutes) was obviously higher in the stomach and small intestine than that in the large intestine and feces, though there is no significant difference (Figure 3, Figure S3 in File S1). In contrast, the relative abundance of Clostridia (belonging to *Firmicutes*) was much higher in the large intestine and feces than that in the small intestine and stomach (*P*<0.05, Figure 3, Figure S3 in File S1). Interesting, the relative abundance of Epsilon-proteobacteria (belonging to Proteobacteria) was much higher in the cecum than that in other sites (*P*<0.05, Figure 3, Figure S3 in File S1). At the family level, anaerobes including Bacteroidaceae (belonging to Bacteroidetes), Prevotellaceae (belonging to Bacteroidetes), Rikenellaceae (belonging to Bacteroidetes), Lachnospiraceae (belonging to Firmicutes) and Ruminococcaceae (belonging to Firmicutes) were enriched in the large intestine and feces (*P*<0.05) while Lactobacillaceae was enriched in small intestine and stomach (Figure 4). A large proportion of unclassified Bacteroidales was no significant difference along the GI tract. At the genus level, large intestine and feces had a higher proportion of *Bacteroides*, *Prevotella*, *Alistipes* (*P*<0.05), while *Lactobacillus* was obviously higher in the stomach and small intestine than that in the large intestine and feces, though there is no significant difference because of large inter-mouse variations (Figure S4 in File S1).

Furthermore, inter-mouse variations were observed from the phylum to the OTU levels: higher inter-mouse variations were detected among the gastric and small intestinal samples than among the large intestinal and fecal samples.

### Clustering of the bacterial community among GI sites

**Figure 5:**
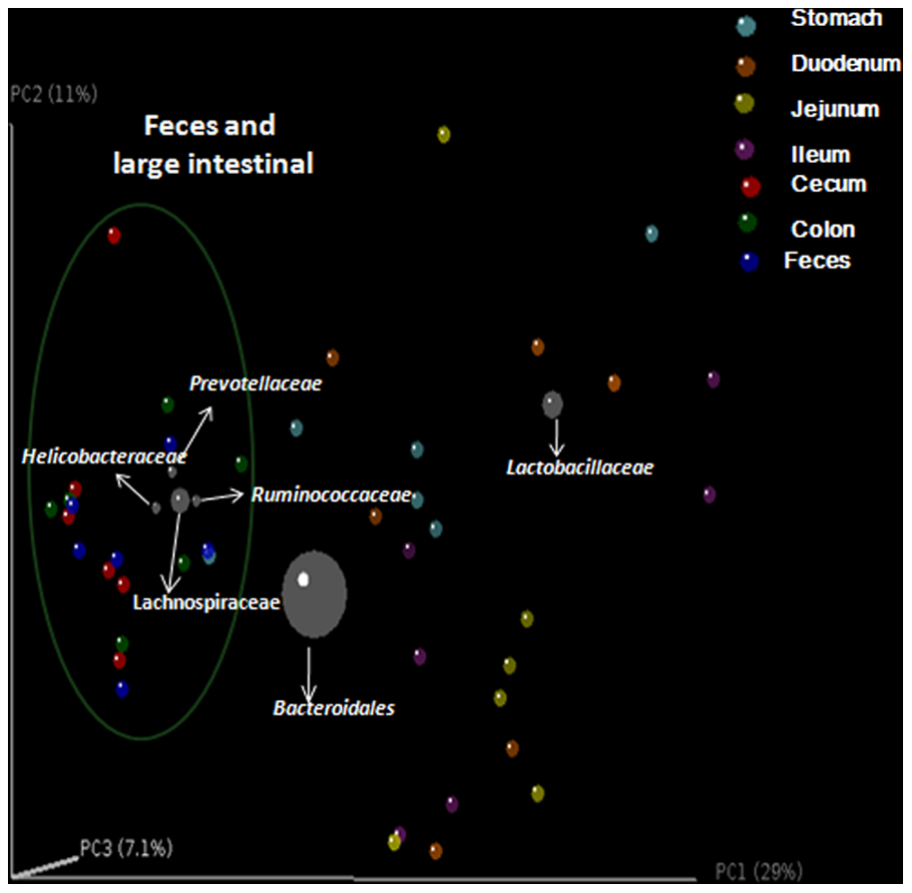
Contribution of different taxonomic groups to separation of samples based on phylogenetic information. The contribution of each group is represented by the size of the circles (grey) overlaid onto a PCoA of unweighted UniFrac distances for all samples within mice digestive tract.

Wards clustering based upon Manhattan distance suggested that the large intestine and fecal bacterial communities were distinct from the gastric and small intestinal ones at the phylum (Figure S5 in File S1), class and family levels (Figure S6 in File S1). Similarly, principal coordinate analysis (PCoA) plots using both weighted (Figure S7 in File S1) and unweighted (Figure 5) UniFrac distances clustered samples mainly by sites, but not by individuals. Bacterial communities in the large intestinal and fecal samples clustered closely to one another while those from gastric and small intestinal samples did not. These results also supported the observation that inter-mouse variations of fecal and large intestinal microbiota were lower than those of gastric and small intestinal samples.

The PCoA plot with the taxonomic information at the family level revealed that the anaerobic Prevotellaceae, Lachnospiraceae, and Rikenellaceae were particularly abundant and important in clustering of fecal and large intestinal microbiota (Figure 5). In contrast, Lactobacillaceae was contributed largely to the community similarity of gastric and small intestinal samples.

### OTUs network across different anatomic sites of the mouse GI tract

**Figure 6:**
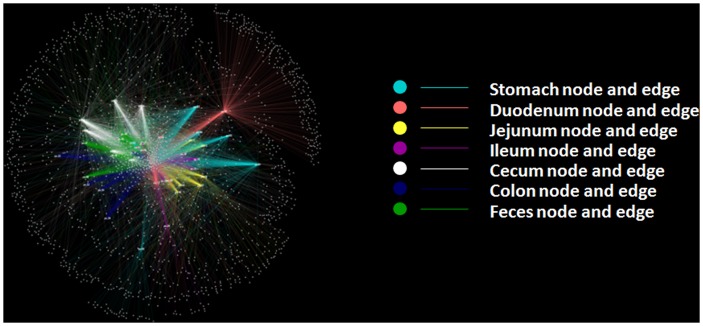
Operational taxonomic unit (OTU) network analysis of bacterial communities from mice GI tract samples for the V3 16S rRNA region.

**Figure 7:**
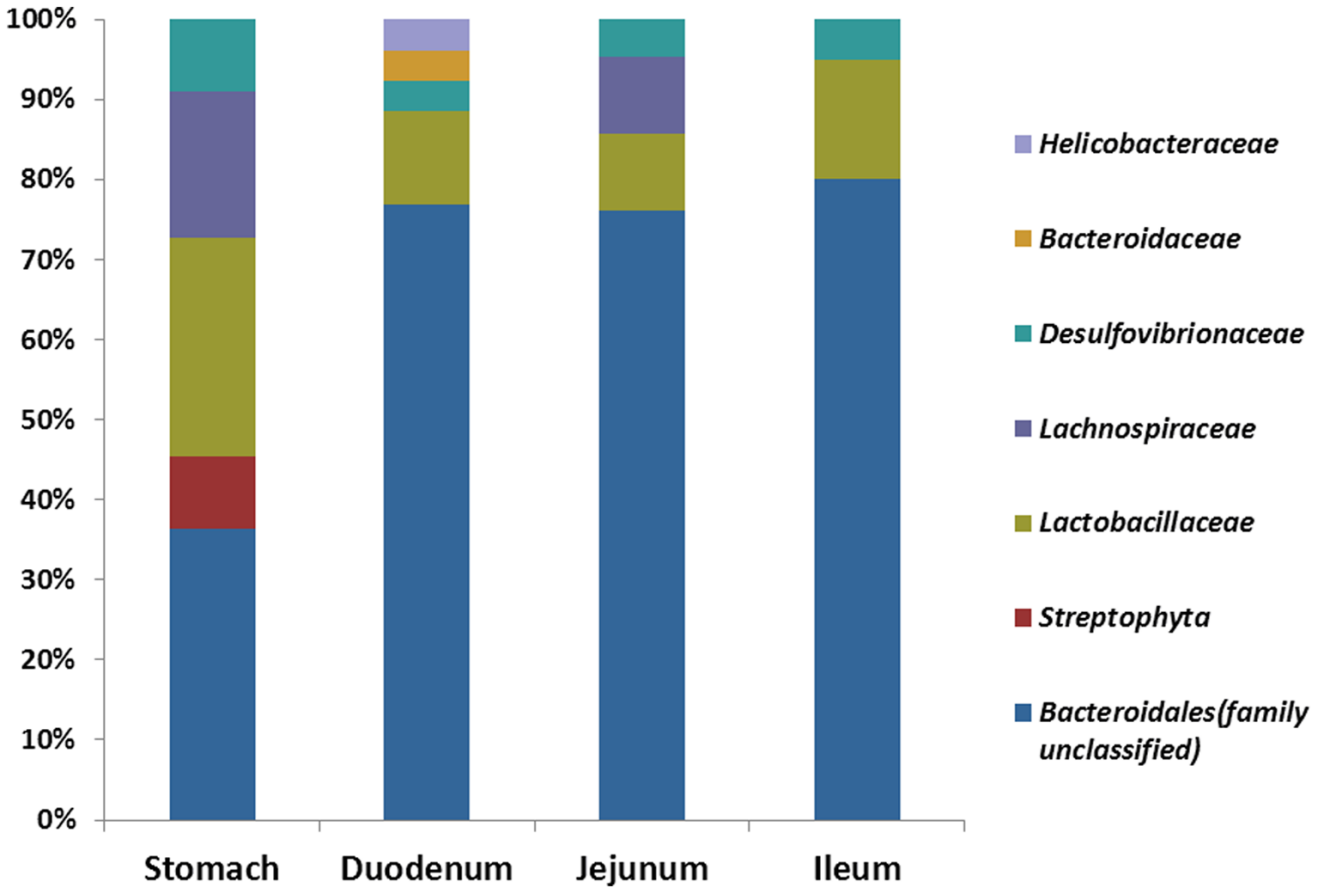
The composition of “core” microbiota of stomach, duodenum, jejunum, and ileum samples.

**Figure 8:**
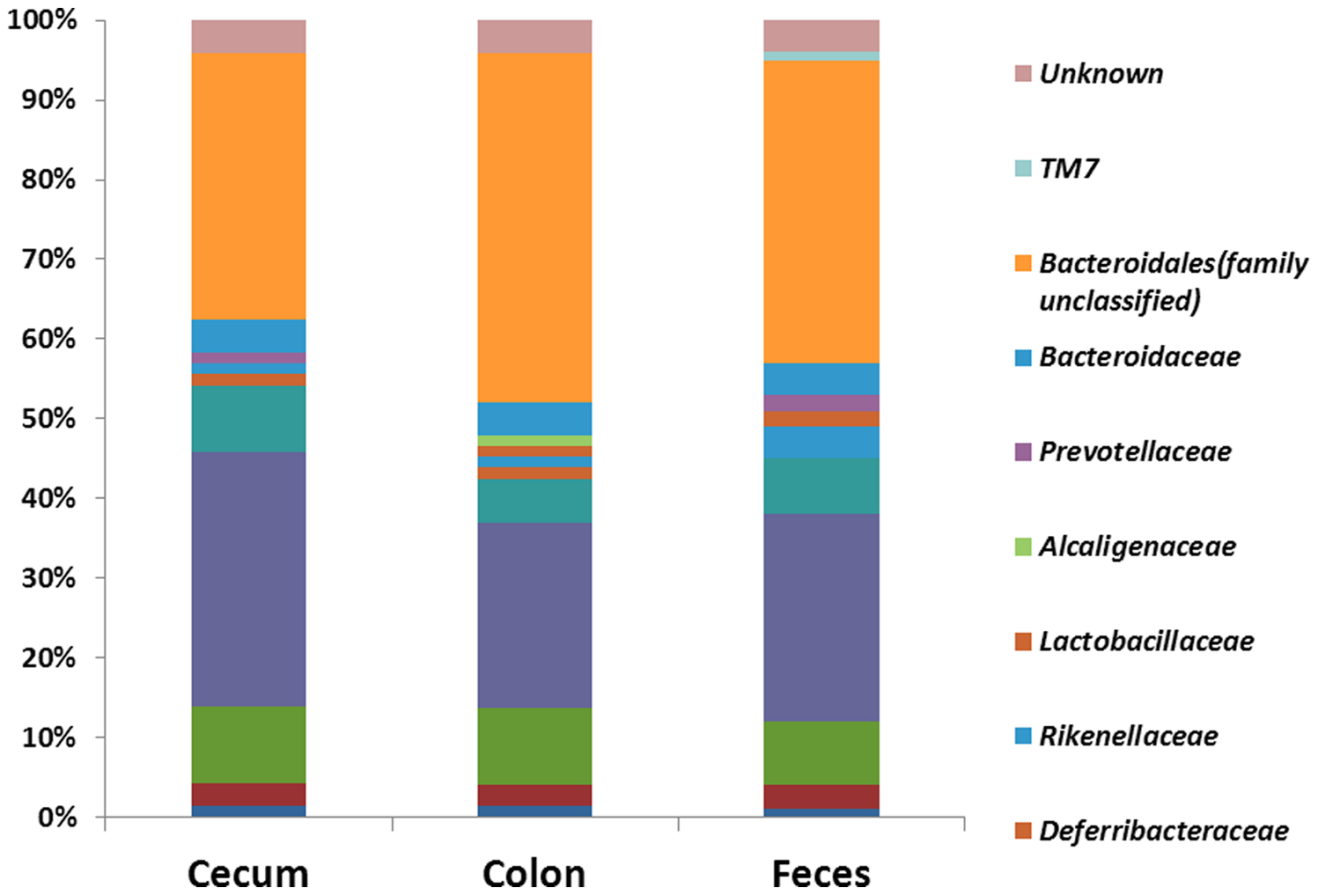
The composition of “core” microbiota of cecum, colon and feces samples.

OTUs and intestine sites (Figure 6) or mice were designated as nodes in bipartite network, in which OTUs were connected to the samples or mice in which their sequences were found. The network-based analyses (Figure 6) showed that samples were more closely associated with one another from the same large intestine sites (cecum and colon) as well as feces than that from same small intestine sites. The results suggested the higher similarity of samples from large intestine and feces than small intestine and stomach. Moreover, the same GI site from different individuals had its “shared” OTUs (Figure S8 in File S1). These shared taxa might perform unique functions to a GI site from other sites. Different sites shared different common “core” microbiota both in amount and composition. The stomachs of the six mouse individuals had a small “core” microbiota (11 OTUs) (Figure S8 in File S1) belonging to Bacteroidales (family unclassified), Lactobacillaceae, Lachnospiraceae, and Desulfovibrionaceae (Figure 7). The duodenum, jejunum and ileum of the six individuals had a relatively bigger “core” microbiota (26, 21 and 20 OTUs) (Figure S8 in File S1), most of which were Bacteroidales (family unclassified), Lactobacillaceae, and Desulfovibrionaceae OTUs (Figure 7). The cecum, colon and feces had the largest “core” microbiota (72, 74 and 84 OTUs) (Figure S8 in File S1), which was composed of bacteria belonging to Bacteroidales (family unclassified), Lachnospiraceae, Ruminococcaceae, Clostridiales, Bacteroidaceae, Prevotellaceae, Rikenellaceae, Deferribacteraceae, Desulfovibrionaceae, Lactobacillaceae, and unclassified bacteria (Figure 8). The more shared OTUs in cecum and colon also indicated more stable microbial communities in large intestine than small intestine.

### Nucleotide sequence accession number

All sequences have been deposited in the GenBank Sequence Read Archive under the accession number SRA061180.

## Discussion

C57BL/6 mice are one of the most common animals used for studying gut microbiota related disease [9]. However, the characteristics and distribution of the microbial community along the C57B/6 mouse GI tract is less clear. Therefore, the investigation into microbiota composition and diversity along the C57BL/6 mouse GI tract was carried out in the present study using a high-throughput pyrosequencing approach.

The overall taxonomic groups represented within the mice GI tract were similar to previous findings. Three bacterial phyla, the Firmicutes, Bacteroidetes and the Proteobacteria dominate the GI tract [18], [19], [20]. However, Ley *et al* [19] reported that there is a dominance of Firmicutes over Bacteriodetes while Caricilli e*t al* [21] reported that Bacteriodetes dominated the WT mice gut. Species difference and diet difference may help explain the apparently different results.

For a long time bacterial diversity along the mammalian GI tract was thought to increase from stomach to feces because the stomach and upper small intestine were viewed as being too harsh (due to the low pH) for microorganisms to grow and to maintain greater diversity. However, our results do not support this traditional concept. Greater diversity was found in both fecal and large intestinal samples as well as duodenal and gastric samples, leaving the least diversity in jejunal and ileal samples (Figure 1). Moreover, duodenal samples contained the most bacterial phyla, although some phyla were detected with very low abundance. In fact, a diverse microbiota was also detected in human and horse stomachs[22], [23], [24]. The detection of diverse microbiota in the stomach and duodenum as well as the varying diversity along the GI tract might be because of the existence and “vanishing” of “transient microbiota”. With food and water from diet, bacteria are continuously ingested from the outer environment to the stomach. During their stay in the host stomach, they are susceptible to death induced by low pH. It is possible that a part of the transient microbiota did quickly escape from the stomach to the duodenum where the neutral pH offers a more conducive environment for bacteria to live than in the stomach. In this study, chow and water were autoclaved before use. However, the SPF environment is not germ free, and the surrounding bacteria may contaminate the diet and water. The large intestine is far from the stomach, receives the least influence from transient microorganisms and offers better surroundings for bacteria to grow. These could be the reasons why the large intestinal and fecal samples had higher and stable PD and SI (Figure 1), as well as the least inter-mouse variations, which is in consistent with the results of Eckburg *et al* [25]. Secondly, many of the bacteria in different phyla may, in fact, be died with their DNA temporarily persevered and therefore detectable with DNA-based approaches, leading to the false positive detection of many bacterial phyla. In this case, the “debris” DNA from the transient microorganisms vanished in the jejunum and ileum, leading to the detection of lower diversity and less bacterial phyla. However, further research is needed to explain these phenomena.

The properties of the different GI sections also exert influences on the microbiota. These include intestinal motility, pH, redox potential, nutrient supplies and host secretions [26], [27], [28]. Since the same GI sections from different individuals have relatively similar physicochemical conditions, the microbiota clustering by GI sections was significant (Figure 5, Figure S7 in File S1). The small intestine is the major site for digestion and the absorption of nutrients, water and electrolytes. The differences between the individual physicochemical conditions in the small intestine are bigger than that in the large intestine [29]. These may be another reason why the gastric and small intestinal microbiota showed remarkable inter-mouse variation, which were also reported in dog and human [30], [31].

The variable dominance of bacteria in different GI sections also supports the influence of GI environment on the microbiota. For example, the oxygen availability of stomach and small intestine were higher, therefore the facultative bacteria including Bacilli (class), Lactobacillaceae (family), *Lactobacillus* (genus) were enriched in stomach and small intestine. In contrast, strictly anaerobic Clostridia (class), Lachnospiraceae (family), Ruminococcaceae (family), Prevotellaceae (family), Rikenellaceae (family), Bacteroidaceae (family) were enriched in the large intestinal and fecal samples where less oxygen is available (Figure 4). But why the relative abundance of Epsilon-proteobacteria (belong to Proteobacteria) was highest in cecum samples remain unclear. However, it is affirmative that fecal samples cannot represent the mouse gut microbiota. Because there are many differences between feces and various gut regions. For example, that epsilon-Proteobacteria are high in the cecum but low in the feces. Therefore, selection of the sampling site along the GI tract is crucially important for the investigation of microbiota-related health and disease issues.

OTU network analyses revealed the existence of a common microbial composition, the “core microbiota”, among the different GI section. Cecum, colon and fecal samples shared more common OTUs, both in terms of numbers and more diverse compositions, than the stomach and small intestine did. These results would support the hypothesis that anatomical regions, which have their own physicochemical conditions, exert important selective pressures on microbiota and play important roles in shaping the GI microbiota. OTU network analyses also revealed that unique microbiota along the GI tract, which could be regarded as the microbial marker of GI sections. It was known that the majority of microbes reside in the gut have a profound influence on human physiology and nutrition, and most of the researches focused on the microbial communities in feces or large intestine. In this study, we found some core OTUs among these samples. Although it was not clear that whether these shared microorganisms were the “permanent residents” or the “passengers” form the foods, the “core microbiota” in the small intestine should be paid more attentions.

In conclusion, the mouse GI tract harbors many distinct niches, each containing a different microbial ecosystem that varies according to the location within the GI tract. Attention should therefore be paid to ensure that the proper GI samples are used to represent each GI microbial community during microbiota-related research.

## Supporting Information

File S1 **Table S1, Figure S1–S8.** Table S1. Overview of pyrosequencing results of each sample. Figure S1 Rarefaction analysis of the different GI sample. Sto:Stomach samples; Duo:Duodenum samples; Jej:Jejunum samples; Ile: Ileum samples; Cec:Cecum samples; Col: Colon samples; Fec: Feces samples. Figure S2 Bacterial families different along the GI tract. ☆ compared VS Cecum *P*<0.05; # compared VS Colon *P*<0.05; compared VS Feces *P*<0.05 Figure S3 Bacterial classes different along the GI tract. ☆ compared VS Cecum *P*<0.05; # compared VS Colon *P*<0.05; compared VS Feces *P*<0.05 Figure S4 Bacterial genus different along the GI tract. ☆ compared VS Cecum *P*<0.05; # compared VS Colon *P*<0.05; compared VS Feces *P*<0.05 Figure S5. Dual hierarchal dendrogram based upon phylum classified using bacterial tag-encoded amplicon pyrosequencing. Sto: Stomach samples; Duo: Duodenum samples; Jej: Jejunum samples; Ile: Ileum samples; Cec: Cecum samples; Col: Colon samples; Fec: Feces samples. The number following the abbreviations stands for the mouse number. For example, Cec1, Cec2, Cec3, Cec4, Cec5, and Cec6 stands for the Cecum sample from the 1^st^, 2^nd^, 3^rd^, 4^th^, 5^th^ and 6^th^ mouse. Figure S6 Dual hierarchal dendrogram based upon class classified using bacterial tag-encoded amplicon pyrosequencing. Sto: Stomach samples; Duo: Duodenum samples; Jej: Jejunum samples; Ile: Ileum samples; Cec: Cecum samples; Col: Colon samples; Fec: Feces samples. The number following the abbreviations stands for the mouse number. For example, Cec1, Cec2, Cec3, Cec4, Cec5, and Cec6 stands for the Cecum sample from the 1^st^, 2^nd^, 3^rd^, 4^th^, 5^th^ and 6^th^ mouse. Figure S7 PcoA Score plot of weighted UniFrac distances for all samples within mice digestive tract. Figure S8 Operational taxonomic unit (OTU) network analysis of bacterial communities from each GI tract site of 6 mice for the V3 16S rRNA region. A, stomach; B, Duodenum; C, Jejunum; D, Ileum; E, Cecum; F, Colon; G, Feces.
(DOC)
