# Supplementary material for: Bacterial Community Mapping of the Mouse Gastrointestinal Tract
Source: PLoS One. 2013 Oct 7;8(10):e74957. doi: 10.1371/journal.pone.0074957 (PMC3792069; doi:10.1371/journal.pone.0074957)
Supplement: File S1 — Table S1, Figure S1–S8. Table S1. Overview of pyrosequencing results of each sample. Figure S1 Rarefaction analysis of the different GI sample. Sto:Stomach samples; Duo:Duodenum samples; Jej:Jejunum samples; Ile: Ileum samples; Cec:Cecum samples; Col: Colon samples; Fec: Feces samples. Figure S2 Bacterial families different along the GI tract. ☆ compared VS Cecum P<0.05; # compared VS Colon P<0.05; compared VS Feces P<0.05 Figure S3 Bacterial classes different along the GI tract. ☆ compared VS Cecum P<0.05; # compared VS Colon P<0.05; compared VS Feces P<0.05 Figure S4 Bacterial genus different along the GI tract. ☆ compared VS Cecum P<0.05; # compared VS Colon P<0.05; compared VS Feces P<0.05 Figure S5. Dual hierarchal dendrogram based upon phylum classified using bacterial tag-encoded amplicon pyrosequencing. Sto: Stomach samples; Duo: Duodenum samples; Jej: Jejunum samples; Ile: Ileum samples; Cec: Cecum samples; Col: Colon samples; Fec: Feces samples. The number following the abbreviations stands for the mouse number. For example, Cec1, Cec2, Cec3, Cec4, Cec5, and Cec6 stands for the Cecum sample from the 1st, 2nd, 3rd, 4th, 5th and 6th mouse. Figure S6 Dual hierarchal dendrogram based upon class classified using bacterial tag-encoded amplicon pyrosequencing. Sto: Stomach samples; Duo: Duodenum samples; Jej: Jejunum samples; Ile: Ileum samples; Cec: Cecum samples; Col: Colon samples; Fec: Feces samples. The number following the abbreviations stands for the mouse number. For example, Cec1, Cec2, Cec3, Cec4, Cec5, and Cec6 stands for the Cecum sample from the 1st, 2nd, 3rd, 4th, 5th and 6th mouse. Figure S7 PcoA Score plot of weighted UniFrac distances for all samples within mice digestive tract. Figure S8 Operational taxonomic unit (OTU) network analysis of bacterial communities from each GI tract site of 6 mice for the V3 16S rRNA region. A, stomach; B, Duodenum; C, Jejunum; D, Ileum; E, Cecum; F, Colon; G, Feces. (DOC) [file pone.0074957.s001.doc]

**Supporting Information**

**Bacterial community mapping of the mouse gastrointestinal tract**

Shenghua Gu^1^, Dandan Chen^1^, Jin-Na Zhang^1, 2^, Xiaomang Lv^3^, Kun Wang^4^, Li-Ping Duan^4^, Xiao-Lei Wu^1*^

**Supplementary tables**

**Table S1 Overview of pyrosequencing results of each sample**

| Sample | Sequence Number | OTU Number | Chao1 | ACE | Good’ s coverage |
| --- | --- | --- | --- | --- | --- |
| Stomach-1 | 8760 | 298 | 460 | 469 | 0.9594 |
| Stomach-2 | 4916 | 158 | 238 | 258 | 0.9663 |
| Stomach-3 | 5245 | 270 | 404 | 415 | 0.9437 |
| Stomach-4 | 12531 | 257 | 347 | 358 | 0.9774 |
| Stomach-5 | 7467 | 248 | 311 | 306 | 0.9660 |
| Stomach-6 | 6344 | 78 | 145 | 138 | 0.9822 |
| Duodenum-1 | 5306 | 534 | 918 | 951 | 0.9066 |
| Duodenum-2 | 5165 | 119 | 162 | 171 | 0.9711 |
| Duodenum-3 | 4876 | 170 | 346 | 292 | 0.9571 |
| Duodenum-4 | 5326 | 199 | 281 | 951 | 0.9503 |
| Duodenum-5 | 6113 | 166 | 245 | 138 | 0.9614 |
| Duodenum-6 | 5319 | 350 | 538 | 564 | 0.9066 |
| Jejunum-1 | 6278 | 139 | 201 | 216 | 0.9671 |
| Jejunum-2 | 4430 | 94 | 147 | 133 | 0.9700 |
| Jejunum-3 | 2640 | 82 | 130 | 120 | 0.9546 |
| Jejunum-4 | 9709 | 172 | 266 | 298 | 0.9865 |
| Jejunum-5 | 6557 | 165 | 244 | 232 | 0.9754 |
| Jejunum-6 | 2414 | 89 | 113 | 116 | 0.9754 |
| Ileum-1 | 6424 | 155 | 220 | 218 | 0.9715 |
| Ileum-2 | 7329 | 180 | 248 | 253 | 0.9735 |
| Ileum-3 | 6353 | 172 | 240 | 242 | 0.9661 |
| Ileum-4 | 5442 | 102 | 135 | 133 | 0.9688 |
| Ileum-5 | 8126 | 99 | 147 | 152 | 0.9851 |
| Ileum-6 | 9034 | 88 | 154 | 134 | 0.9881 |
| Cecum-1 | 4379 | 251 | 444 | 431 | 0.9305 |
| Cecum-2 | 6896 | 218 | 297 | 306 | 0.9642 |
| Cecum-3 | 6627 | 296 | 450 | 446 | 0.9486 |
| Cecum-4 | 6830 | 276 | 400 | 426 | 0.9480 |
| Cecum-5 | 6539 | 238 | 310 | 336 | 0.9551 |
| Cecum-6 | 8058 | 329 | 591 | 509 | 0.9505 |
| Colon-1 | 7653 | 137 | 185 | 175 | 0.979 |
| Colon-2 | 7629 | 182 | 248 | 247 | 0.979 |
| Colon-3 | 6593 | 177 | 201 | 203 | 0.977 |
| Colon-4 | 8096 | 185 | 265 | 248 | 0.974 |
| Colon-5 | 7751 | 201 | 268 | 256 | 0.967 |
| Colon-6 | 7854 | 205 | 313 | 248 | 0.973 |
| Feces-1 | 10888 | 306 | 445 | 429 | 0.968 |
| Feces-2 | 2886 | 178 | 242 | 246 | 0.933 |
| Feces-3 | 10052 | 374 | 549 | 553 | 0.959 |
| Feces-4 | 9120 | 263 | 401 | 407 | 0.978 |
| Feces-5 | 6852 | 350 | 566 | 536 | 0.940 |
| Feces-6 | 6427 | 226 | 294 | 290 | 0.967 |

**Supplementary Figures**


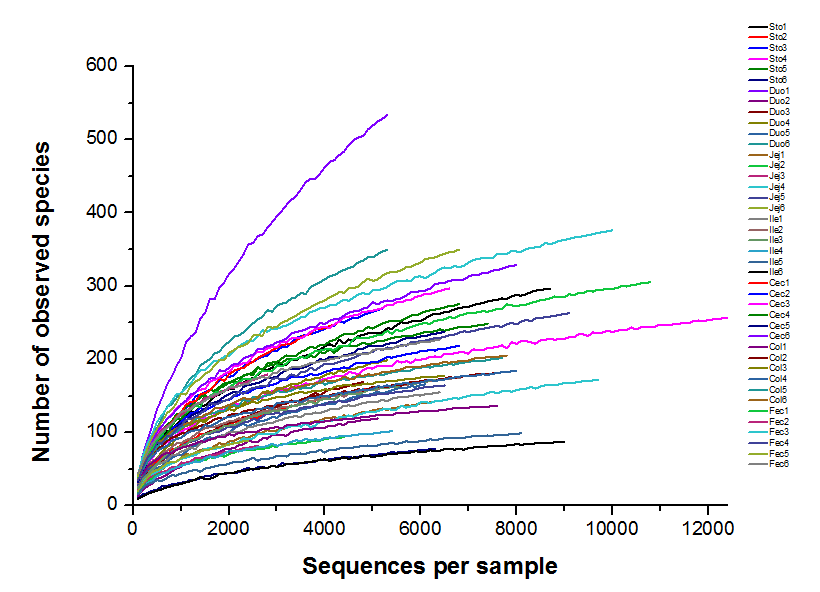


**Figure S1** Rarefaction analysis of the different GI sample. Sto:Stomach samples; Duo:Duodenum samples; Jej:Jejunum samples; Ile: Ileum samples; Cec:Cecum samples; Col: Colon samples; Fec: Feces samples


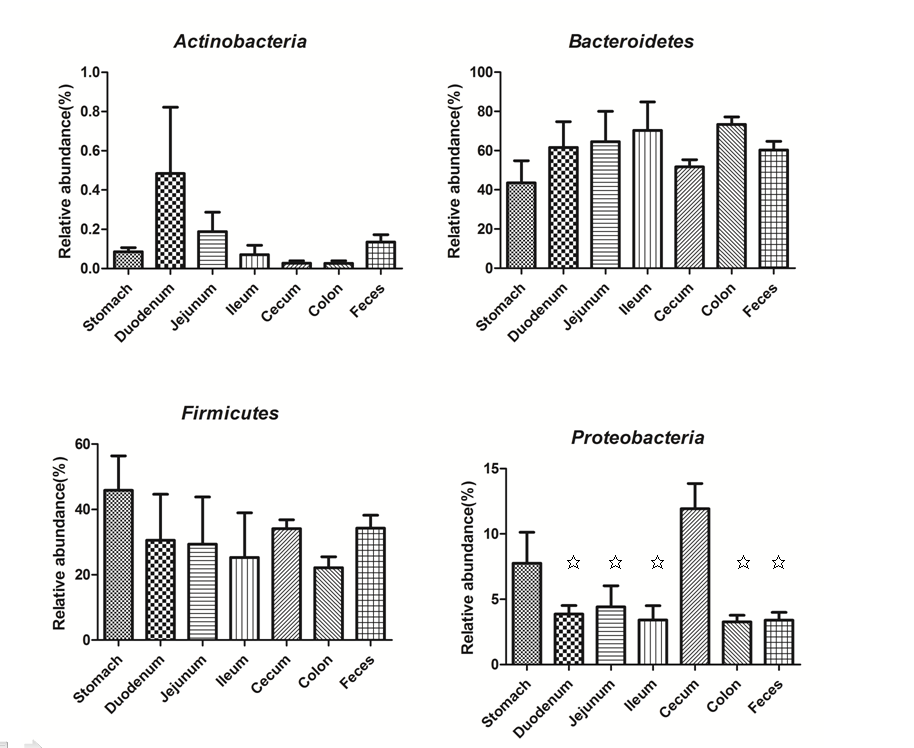


**Figure S2** Bacterial families different along the GI tract.

☆ compared VS Cecum *P*<0.05; ﹟ compared VS Colon *P*<0.05 ;※ compared VS Feces *P*<0.05


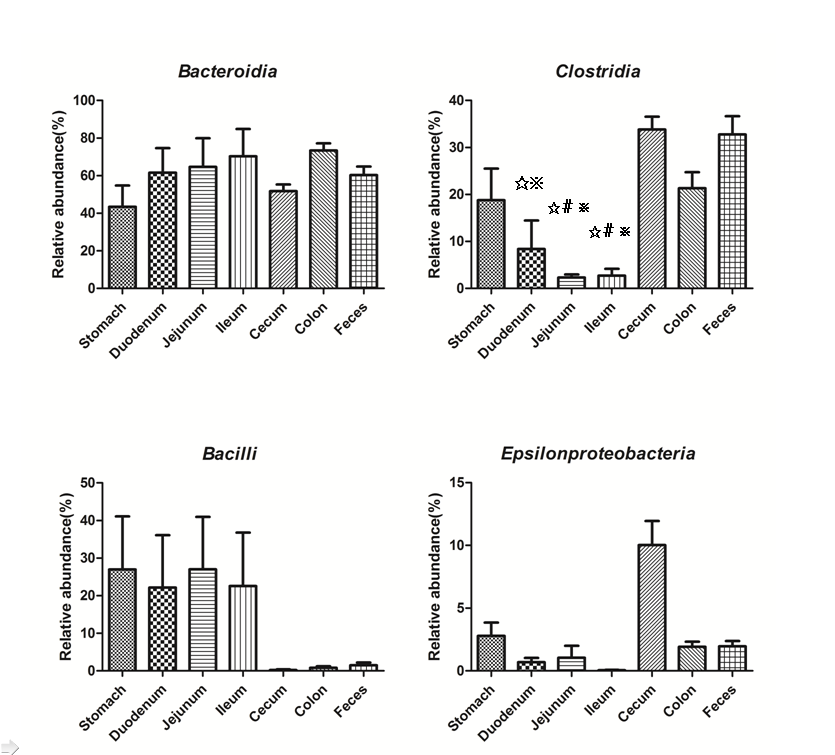


**Figure S3** Bacterial classes different along the GI tract.

☆ compared VS Cecum *P*<0.05; ﹟ compared VS Colon *P*<0.05 ;※ compared VS Feces *P*<0.05


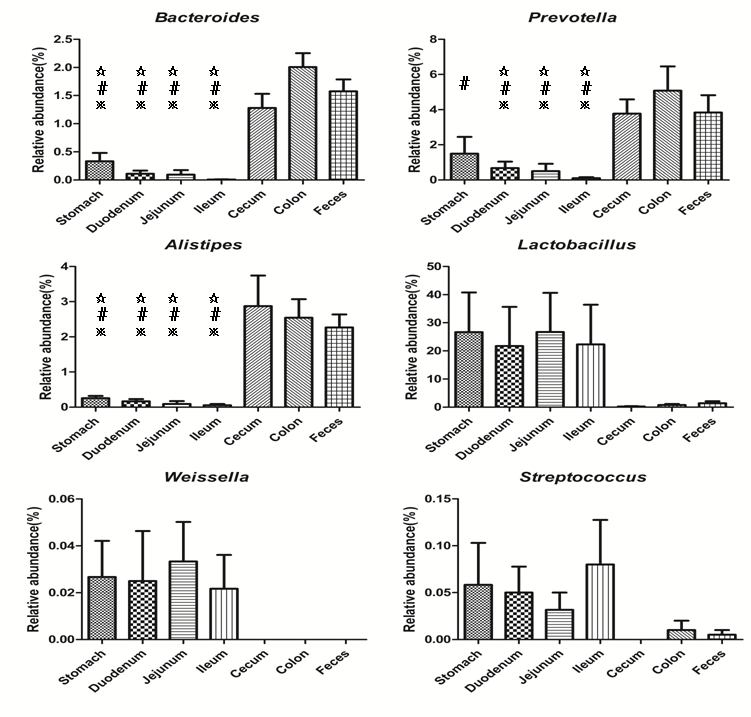


**Figure S4** Bacterial genus different along the GI tract.

☆ compared VS Cecum *P*<0.05; ﹟ compared VS Colon *P*<0.05 ;※ compared VS Feces *P*<0.05


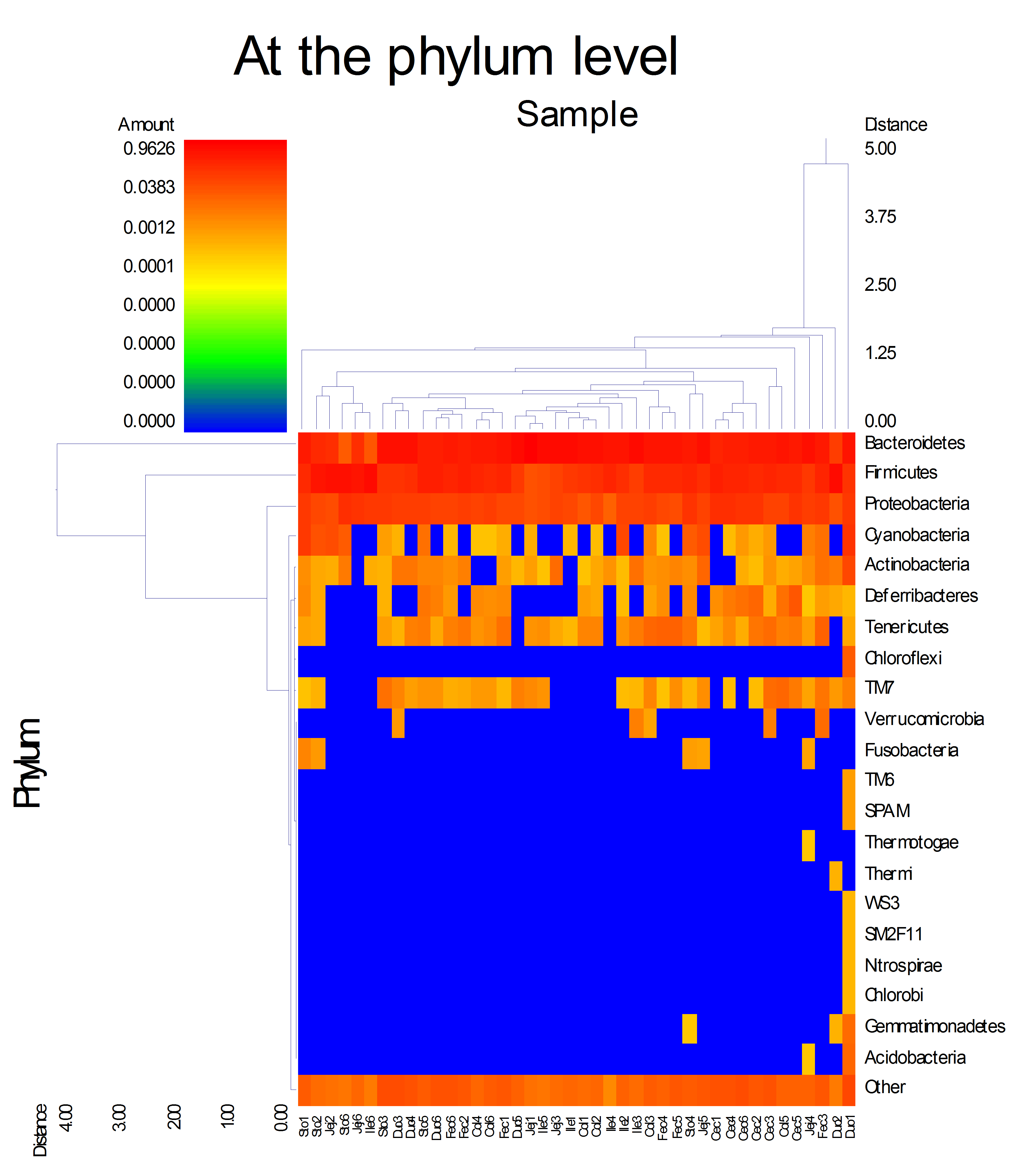


**Figure S5. Dual hierarchal dendrogram based upon phylum classified using bacterial tag-encoded amplicon pyrosequencing.**

Sto: Stomach samples; Duo: Duodenum samples; Jej: Jejunum samples; Ile: Ileum samples; Cec: Cecum samples; Col: Colon samples; Fec: Feces samples. The number following the abbreviations stands for the mouse number. For example, Cec1, Cec2, Cec3, Cec4, Cec5, and Cec6 stands for the Cecum sample from the 1^st^, 2^nd^, 3^rd^, 4^th^, 5^th^ and 6^th^ mouse.

**Figure S6** Dual hierarchal dendrogram based upon class classified using bacterial tag-encoded amplicon pyrosequencing.

Sto: Stomach samples; Duo: Duodenum samples; Jej: Jejunum samples; Ile: Ileum samples; Cec: Cecum samples; Col: Colon samples; Fec: Feces samples. The number following the abbreviations stands for the mouse number. For example, Cec1, Cec2, Cec3, Cec4, Cec5, and Cec6 stands for the Cecum sample from the 1^st^, 2^nd^, 3^rd^, 4^th^, 5^th^ and 6^th^ mouse.


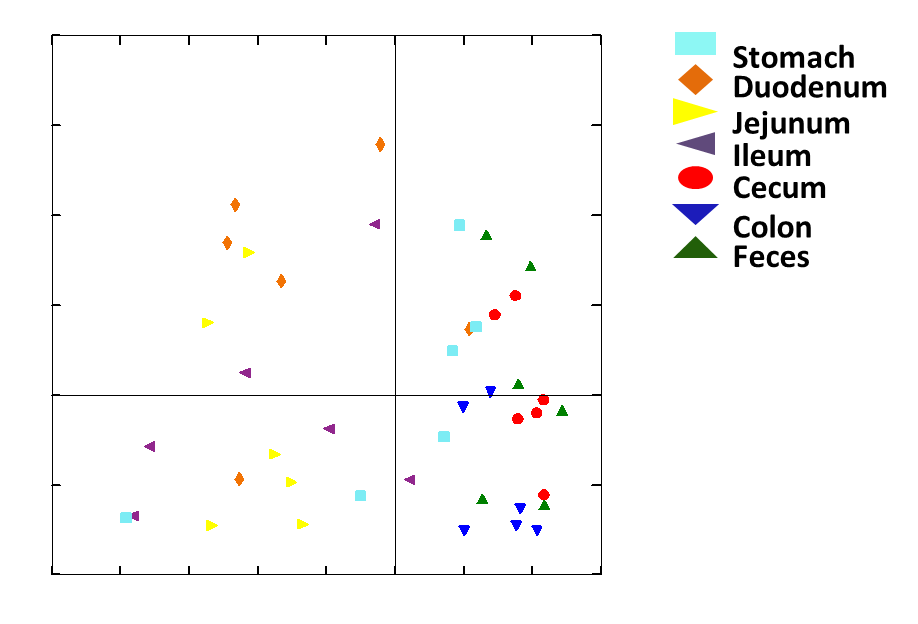


**Figure S7** PcoA Score plot of weighted UniFrac distances for all samples within mice digestive tract.


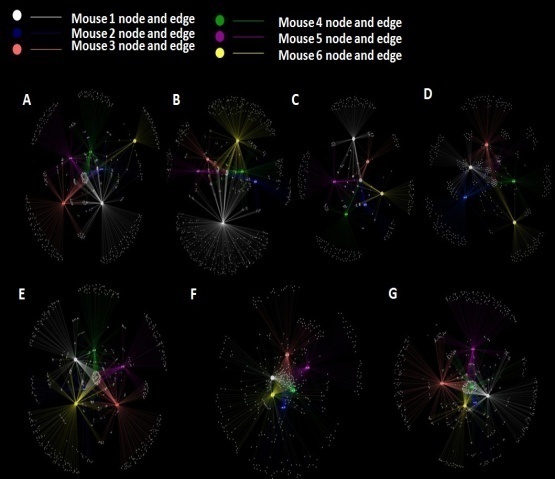


**Figure S8** Operational taxonomic unit (OTU) network analysis of bacterial communities from each GI tract site of 6 mice for the V3 16S rRNA region. A, stomach; B, Duodenum; C, Jejunum; D, Ileum; E, Cecum; F, Colon; G, Feces.
